# Restoration of a Peg Lateral Incisor Using the Putty Index Technique: A Case Report

**DOI:** 10.7759/cureus.68964

**Published:** 2024-09-08

**Authors:** Shwetana Kurundkar, Aditya Patel, Anuja Ikhar, Manoj Chandak, Neha K Urkande, Pratik Rathod, Priyanka R Bhojwani

**Affiliations:** 1 Department of Conservative Dentistry and Endodontics, Sharad Pawar Dental College and Hospital, Datta Meghe Institute of Higher Education and Research, Wardha, IND

**Keywords:** anterior teeth restoration, direct restoration, peg lateral incisor, putty index technique, resin based composite

## Abstract

The restoration of peg lateral incisors poses considerable aesthetic and functional issues. This case report describes the use of a putty index approach for conservatively restoring peg-shaped maxillary lateral incisors with resin composites. A 21-year-old female patient with good periodontal health and appropriate occlusal interactions had treatment at the Department of Conservative Dentistry and Endodontics. The restoration method included initial impressions, shade selection, etching, bonding, and gradual composite application, followed by curing and polishing. The putty index was used as a reference to accurately recreate the palatal enamel. This approach provides a low-cost, minimally intrusive treatment that maintains natural tooth structure while producing satisfactory aesthetic outcomes. Direct adhesive resin restorations are being highlighted as a feasible option, as technology and bonding techniques progress. This case shows the significance of modifying treatment plans based on patient characteristics and practitioner expertise.

## Introduction

As composites are widely employed materials for the restoration of anterior teeth abnormalities, a dentist must possess significant expertise regarding the usage of this material [1]. Creating and replicating the correct contour and contact form is an essential challenge in anterior composite restorations. Matrix application can be crucial for successful anterior composite restorations [2]. Hypodontia is the result of abnormalities that occur throughout the beginning or propagation of the tooth bud-tooth-forming process [3]. Variations in size are most frequently caused by anomalies in the morphology of the tooth, resulting in late perturbations during the process of differentiation [4].

Maxillary lateral incisors that are peg-shaped or smaller than normal in the mesiodistal dimension show different degrees of trait expression, even though the particular gene(s) responsible for hypodontia have not been known [5]. A peg lateral has been termed a "tapered, undersized maxillary lateral incisor" and can be linked to developmental defects like retained deciduous teeth and canine transposition [6]. The central incisor moves distally in those with faulty lateral incisors, resulting in a midline diastema [4]. The emergence of diastema in the anterior teeth can be caused by smaller and malformed lateral incisors [6]. Unless there are additional congenital causes or habits, these patients may have generally normal dentitions. The prevalence of additional tooth developmental anomalies is lower than that of peg-shaped maxillary lateral incisors, as given in reports. In a study conducted by Bäckman and Wahlin, incisors with a peg shape were observed in 0.8% of 739 children [7]. Another study reported a prevalence of 0.4% [8]. The emergence of permanent tooth discrepancies and hypodontia was reported to occur in 4% of cases [9]. Maxillary lateral incisors that are peg-shaped have been linked to various developmental defects in some prior studies, one of which found a higher occurrence on the left side of the maxilla [10].

Several considerations must be made while restoring peg-shaped lateral incisors, according to the patient's expectations and the practitioner’s skill [11]. Canine position, the need for extractions, the need for functional and aesthetic needs, and the possibility of combining orthodontic and restorative care can be considered when choosing the course of treatment [12]. Possible treatment options for peg lateral incisors are extraction of the peg-shaped lateral incisor followed by orthodontic treatment to move the canine into the lateral incisor space; canine recontouring to resemble lateral incisors; extraction of the tooth and replacement with a single-tooth implant-supported restoration or a fixed partial denture; or restoring the peg-shaped lateral incisors directly or indirectly to achieve normal tooth morphology [11-13]. These treatment modalities could lead to satisfactory outcomes [6]. The composite restoration of peg lateral incisors using the indirect injection molding technique is a recent advancement that offers improved precision and aesthetics compared to traditional methods. The key innovations include better control over tooth morphology, minimizing chair time, and achieving more predictable, uniform restorations. It fills a knowledge gap by providing a less invasive, highly reproducible method with improved aesthetic outcomes, especially for challenging cases like peg lateral incisors, where maintaining natural shape and symmetry is crucial. This clinical report outlines a straightforward indirect method for enhancing the aesthetic appearance of peg-shaped lateral incisors.

## Case presentation

**Figure 1:**
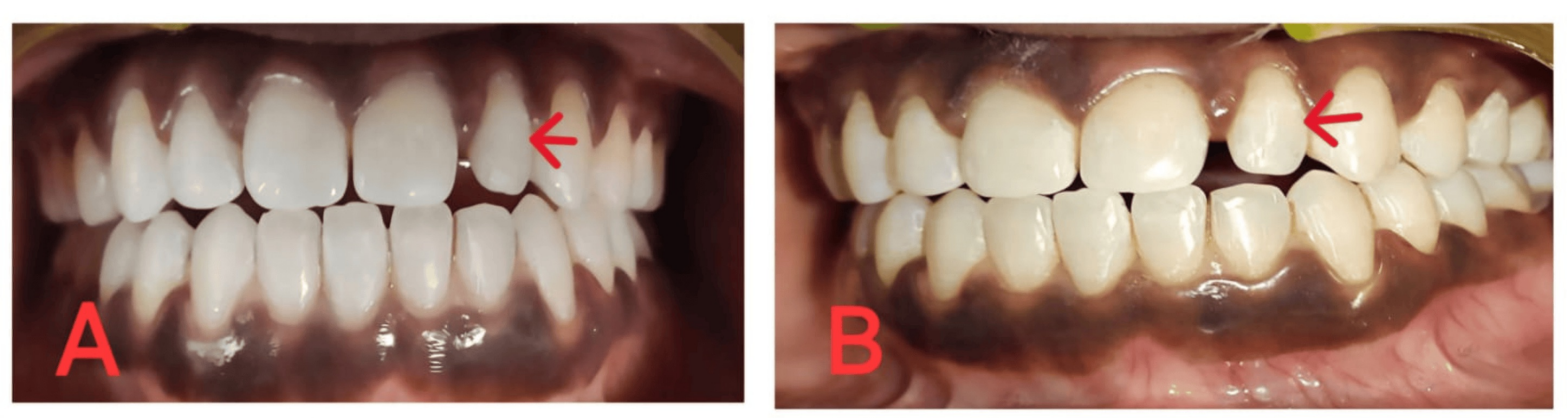
Peg-shaped lateral incisor with respect to 22

A 21-year-old female patient reported to the Department of Conservative Dentistry and Endodontics. On examination, the patient had normal, healthy gingiva and a class I interocclusal molar relationship (Figure 1).

**Figure 2:**
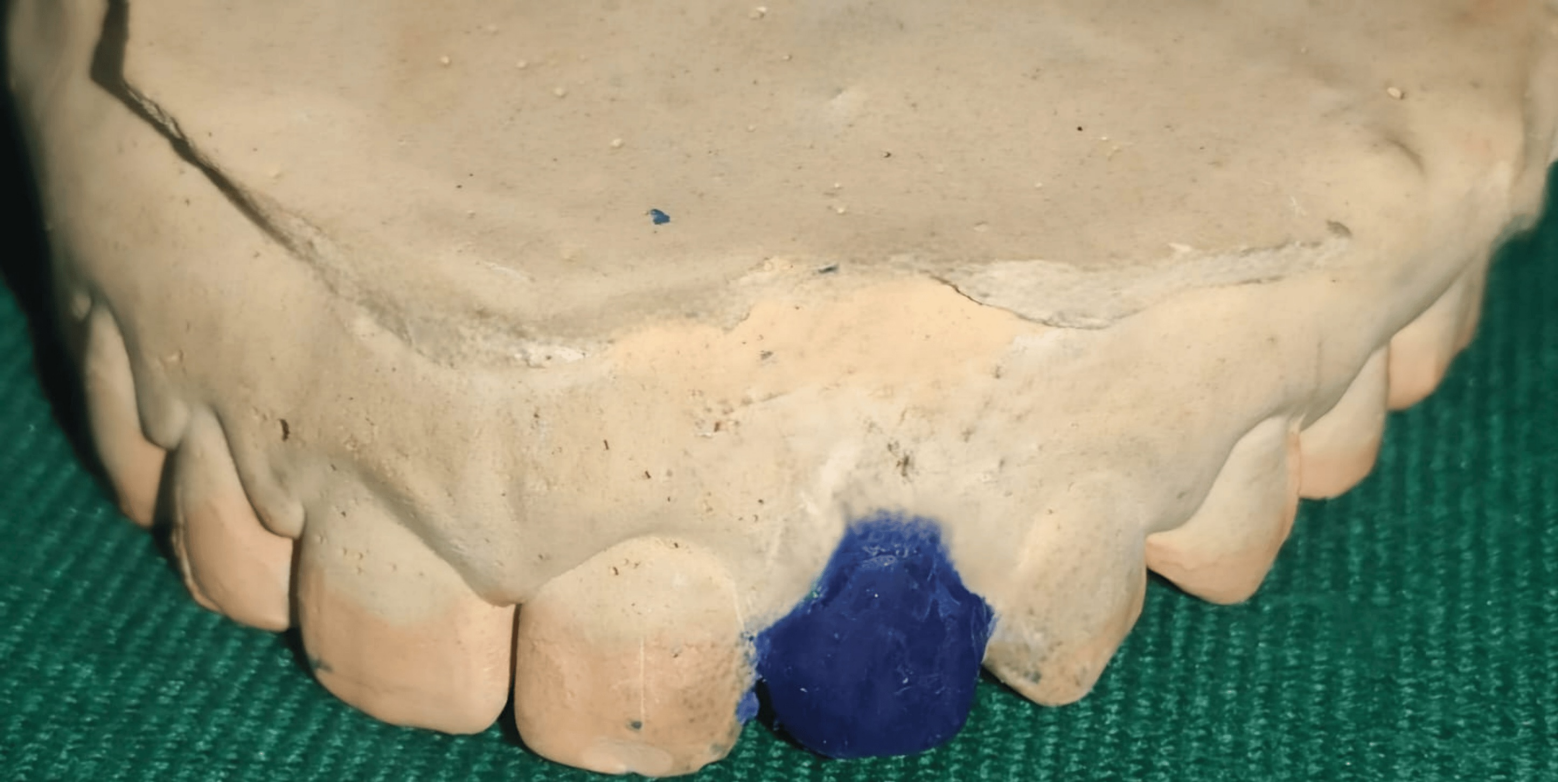
Study model with wax buildup

**Figure 3:**
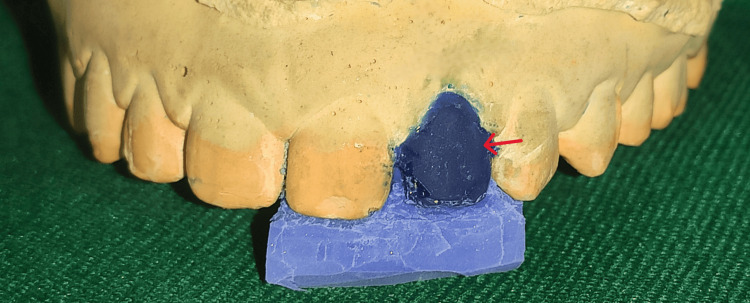
Putty index on study model

A primary impression was made with dental alginate (Dentsply Sirona Inc., Charlotte, NC, United States), after which a diagnostic cast was created. A diagnostic wax-up was done on the cast with the help of inlay wax (Figure 2), and a putty index was made (Figure 3).

**Figure 4:**
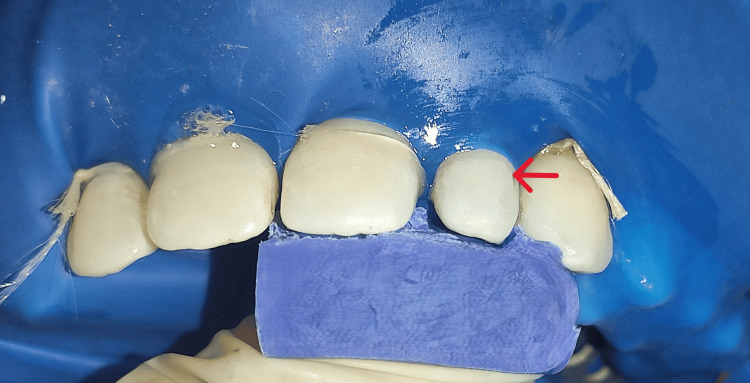
Putty index in patient's tooth

Shade selection was done using the button technique. The putty index was then divided into two parts in the mesiodistal direction to obtain palatal and labial halves, respectively. The palatal half was then inserted intraorally and checked for proper fit, serving as a reference guide and a rigid plate for reconstructing palatal enamel later on (Figure 4).

**Figure 5:**
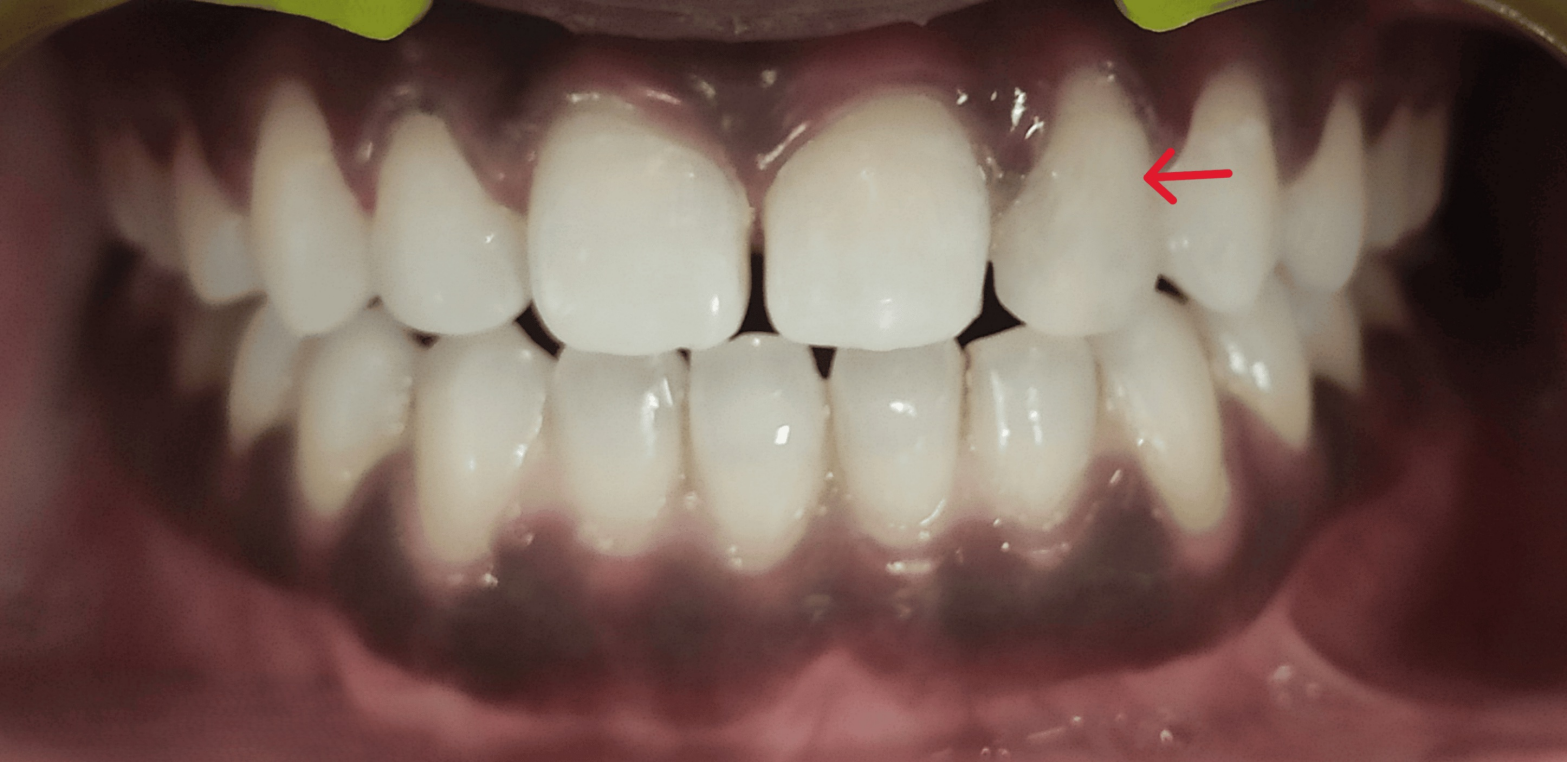
Resin-based composite restoration with respect to 22

The selection of an appropriate shade of direct composite material (Spectrum, Dentsply) was made. After isolation, the lateral incisor's labial and palatal surfaces were etched for 15 seconds using 37% phosphoric acid, and a bonding agent was then applied. The restoration was completed with a resin composite (Spectrum, Dentsply) using an incremental technique (Figure 5).

**Figure 6:**
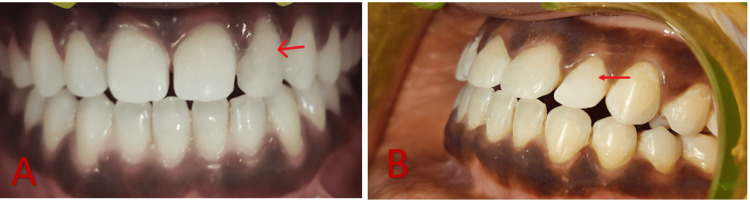
Follow-up after six months

The restoration was cured using a curing light. Polishing discs were used for contouring and polishing the resin composite restoration. Interproximal finishing was done with the help of finishing strips. Postoperative instructions were given to the patient, and she was scheduled for recall at six-month intervals (Figure 6).

## Discussion

Patients with peg lateral incisors have aesthetic problems related to both the tooth's morphological defect and the gaps between the teeth. Treatment aims primarily to restore or replace underdeveloped teeth and minimize the gaps. For patients who do not smoke or consume dark-colored beverages (which could stain teeth), restoration with resin composite may be the first choice for various reasons, as it preserves natural tooth structure, often doesn't require local anesthesia, can be completed in a single visit, and is comparatively cost-effective [6].

Walls et al. [13] utilized resin composite laminate veneers to conceal discolored teeth or hypoplasia in the maxillary front teeth of 68 patients. This method resulted in significant improvements in both aesthetics and functionality over two years. The clinical study demonstrated that the periodontal health of patients' teeth notably progressed from the initial assessment to the application of the veneers. Moreover, patients who struggled with maintaining good oral hygiene showed adverse effects on gingival health after receiving the veneer restorations. Additionally, there were instances where the periodontal score was associated with discontinuities in the veneer’s gingival margin, though no overall correlation was found between gingival status and marginal discontinuity across the sample.

The therapeutic approach for the mentioned patient involved preserving the peg-shaped lateral incisor of the maxillary arch and restoring its sound tooth structure using a bonded composite. This conservative approach was selected to preserve the tooth form. Resin composite restorations demonstrate excellent physical properties, marginal integrity, and aesthetics [14]. For peg-shaped lateral incisors, direct adhesive resin restorations are both cost-effective and can be completed chairside. Advances in technology and bonding systems have further increased the success rate of these restorations [2]. From an aesthetic perspective, managing anterior tooth mutilation is a significant challenge for clinicians. Considering the patient’s socioeconomic status and age, a direct restoration using the putty matrix technique was planned in this case. In addition to serving as a matrix, it also functions as a rigid template, which acts as a wall to support the resin-based composite. It helps determine the thickness of the incisal edge and the cervico-incisal length, facilitating easier placement into the area that requires restoration [2]. 

## Conclusions

When compared to other invasive aesthetic procedures, the putty index matrix approach is a quick, easy, and affordable process. The matrix also serves as a guide to restore the teeth's lost anatomical structure and contour. This technique can be utilized for restoring both a single tooth and multiple teeth. Clinicians of any experience level can employ this systematic approach and achieve excellent results.
